# Chloroplast genome analysis and phylogenetic position of *Polygonatum sinopubescens* and comparison with related species

**DOI:** 10.1371/journal.pone.0338103

**Published:** 2025-12-05

**Authors:** Zhong-Mei Mo, Chun-Mou Wei, Hai-Ying Yu, Chuan-Dong Yang

**Affiliations:** 1 College of A&F Engineering and Planning, Tongren University, Tongren, Guizhou, China; 2 College of Agriculture, Tongren Polytechnic University, Tongren, Guizhou, China; Nuclear Science and Technology Research Institute, IRAN, ISLAMIC REPUBLIC OF

## Abstract

The genus *Polygonatum* (Asparagaceae) comprises perennial herbaceous plants with significant economic and medicinal value. In this study, we analyzed the complete chloroplast (cp) genome of *Polygonatum sinopubescens* and compared it with closely related species. The primary objective was to elucidate structural variations, species divergence, and phylogenetic relationships among taxa. The cp genome of *P. sinopubescens* exhibits the typical quadripartite structure, consisting of a large single-copy (LSC) region, a small single-copy (SSC) region, and a pair of inverted repeats (IRs), with a total sequence length of 155,307 bp and a GC content of 37.68%. The present analysis revealed a high degree of consistency in gene order and GC content between *P. sinopubescens* and other *Polygonatum* species. A total of 112 genes were annotated, including 78 protein-coding genes, 30 tRNA genes, and 4 rRNA genes. The genome contained 67 simple sequence repeats (SSRs), and codon usage was biased toward codons ending in A/T; among the 30 codons with RSCU > 1, 93.3% ended with A/T. Nucleotide polymorphism analysis identified nine highly variable regions, and selection pressure analysis revealed that only *ndh*A, *ycf*2, *acc*D, and *rbc*L genes were under positive selection (Ka/Ks > 1), which was observed in only a subset of species. Phylogenetic analyses indicated that *Polygonatum* is a monophyletic group that can be divided into three major clades. *P. sinopubescens* was placed in sect. *Polygonatum* and was most closely related to *P. filipes*. This study provides a comprehensive characterization of the cp genome of *P. sinopubescens* and clarifies its phylogenetic placement, offering important references for species identification, evolutionary studies, and phylogenetic research within *Polygonatum*.

## 1. Introduction

The genus *Polygonatum* Mill. (Asparagaceae) comprises perennial herbaceous plants, with approximately 39 species distributed in China [1]. “Polygonati Rhizoma”is a medicinal and edible plant known for its pharmacological properties, including anti-aging, anti-tumor, hypoglycemic, and immune-enhancing effects [2–4]. Due to its high medicinal value, over a hundred commercial pharmaceutical and healthcare products are derived from *Polygonatum* [5]. Based on morphological, palynological, cytological, and molecular biological studies [6–10], recent research has classified *Polygonatum* into three sections: section *Verticillata*, section *Polygonatum*, and section *Sibirica* [11–13]. The Chinese Pharmacopoeia primarily records the dried rhizomes of *Polygonatum odoratum*, *Polygonatum sibiricum*, *Polygonatum kingianum*, and *Polygonatum cyrtonema* as medicinal ingredients [14]. However, due to morphological similarities among *Polygonatum* species, botanical identification is often challenging, leading to frequent adulteration in the market [15]. Common adulterants include *Polygonatum zanlanscianense*, *Polygonatum cirrhifolium*, *Polygonatum verticillatum*, and other species from section *Verticillata* [16,17]. These adulterants generally exhibit inferior medicinal properties, and some may compromise clinical safety and efficacy [18,19]. Therefore, effective identification methods are needed to ensure the quality of Polygonati Rhizoma medicinal materials.

The chloroplast (cp) is a crucial organelle for photosynthesis and energy conversion in plant cells. In angiosperms, the cp genome is maternally inherited and is characterized by structural stability, conserved coding sequences, and rich genetic information, making it a valuable resource for species identification and genetic variation studies [20]. Most higher plants possess a typical quadripartite chloroplast genome structure, comprising a large single-copy (LSC) region, a small single-copy (SSC) region, and a pair of inverted repeats (IRs) [21]. With the advent of high-throughput sequencing technologies, cp genomes have been widely employed in plant phylogenetics, species identification, genetic diversity analysis, and genetic engineering [22–24]. The complete chloroplast genome, used as a super-barcode, has shown great potential in the identification of medicinal plants. Wu et al. [25] applied this technique to successfully distinguish *Fritillaria* species recorded in the Chinese Pharmacopoeia from their close relatives and adulterants. This technology has also been widely applied in other medicinal plants and has achieved good results [26]. For example, Cui et al. [27] accurately identified three closely related species of *Amomum* (*A. villosum*, *A. villosum* var. *xanthioides*, and *A. longiligulare*). Similarly, Zhu et al. [28] confirmed that the complete chloroplast genome dataset provides the strongest discriminating power for *Dendrobium officinale* and its related species. Chen et al. [29] demonstrated that the chloroplast genome not only enables precise identification of *Thalictrum fargesii*, but that its highly variable regions (such as *ndh*D-*psa*C and *rpl*16-*rps*3) also hold promise for developing specific molecular markers for identifying ethnomedicines and their contaminants. In addition, this technique has been successfully applied to identify *Mussaenda pubescens* [30], *Sophora tonkinensis* [31], members of the subfamily *Aroideae* [32], and to determine their phylogenetic positions. Wang et al. [33] combined chloroplast genome and internal transcribed spacer(ITS) sequences to confirm that *C. × ventricosum* is most closely related to *C. calceolus* and supportsd its origin as an interspecific hybrid between *C. calceolus* and *C. macranthos*. Similarly, studies on the genus *Lasianthus* not only clarified phylogenetic relationships using the complete chloroplast genome but also identified an efficient identification marker composed of ITS2 + psaI-ycf4 [34].

Therefore, utilizing the cp genome for species identification within *Polygonatum* can enhance the safety and efficacy of medicinal applications and promote the sustainable development of *Polygonatum* resources. *Polygonatum sinopubescens* is an endemic species discovered in Yinjiang, Guizhou Province [35]. Morphologically, *P. sinopubescens* is distinguished from *Polygonatum filipes* by its densely hairy stems (approximately 30 cm tall), petioles with soft hairs, young leaves densely covered with short hairs on the abaxial surface, inflorescences bearing 2 ~ 3 flowers per peduncle, pedicels covered with long soft hairs, filaments measuring 7 ~ 11 mm in length with pubescent upper portions, and obovoid berries, These characteristics classify *P. sinopubescens* within sect. *Polygonatum* of *Polygonatum* [36]. Nutritional composition analysis has further confirmed that *P. sinopubescens* is a high-quality functional plant resource with both medicinal and edible applications [37]. This study reports the complete chloroplast genome of *P. sinopubescens*, expanding the genomic resources for *Polygonatum* and providing a valuable reference for species classification, genetic diversity research, and medicinal applications.

## 2. Materials and methods

### 2.1. Plant materials

**Fig 1:**
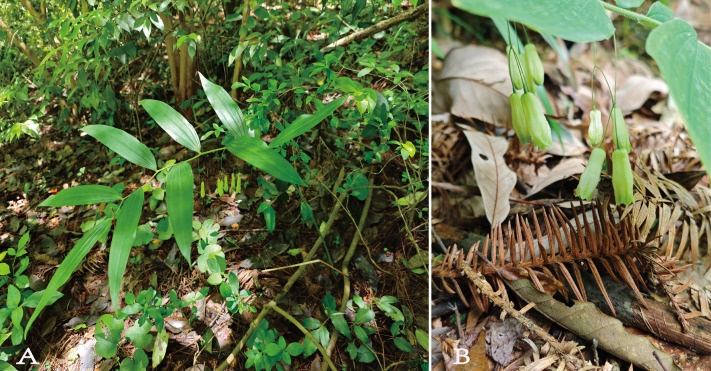
Photographs of *Polygonatum sinopubescens* (A) Habitat, (B) Inflorescence.

The plant samples were collected from Yinjiang Tujia Autonomous County, Tongren City, Guizhou Province (27°43’1.98“N,108°28’15.21” E). They were identified as *P. sinopubescens* of the *Polygonatum* genus by Professor Yang Chuandong of Tongren University (Fig 1). The fresh leaves collected were stored in dry ice and sent to Qingke Company for DNA extraction and third-generation chloroplast gene sequencing.

### 2.2. Chloroplast DNA extraction

Fresh young leaves of the *P. sinopubescens* sample were frozen in liquid nitrogen, and high-quality genomic DNA was extracted using a modified cetyltrimethylammonium bromide (CTAB) method. The DNA concentration was measured using the Thermo Scientific NanoDrop software, and DNA quality was assessed by 1% agarose gel electrophoresis. An Illumina genomic library was constructed and subjected to 2 × 150 bp sequencing using the NovaSeq X Plus platform (Illumina, San Diego, CA, USA) at Qingke Biotechnology (Beijing, China).

### 2.3. Chloroplast DNA sequencing, assembly and data processing

For chloroplast genome assembly, clean data were processed using GetOrganelle v1.7.5 [38], with the seed database as a reference. Genome assembly was performed using SPAdes, and the assembly order of chloroplast contigs was verified by alignment against the NT database. Contigs with consistent sequence order were selected as the final genome assembly. The starting position and orientation of the chloroplast genome sequence were determined based on a reference genome, along with the identification of possible partition structures (LSC/IR/SSC), resulting in the finalized chloroplast genome sequence. Gene annotation, including predictions of protein-coding genes, tRNA genes, and rRNA genes, was performed using GeSeq, with manual correction of gene boundaries and exon/intron junctions. The circular genome map was visualized using OGDRAW [39]. The final annotated chloroplast genome was submitted to NCBI, and the registration number PQ858224 was obtained.

### 2.4. Comparative bioinformatic analysis

The relative synonymous codon usage (RSCU) values were calculated using the Cusp software (EMBOSS v6.6.0.0) to determine codon preference. Microsatellite loci were analyzed using the MISA software (version 1.0), with parameters set to ≥10 repeats for mononucleotides, ≥ 5 repeats for dinucleotides, ≥ 4 repeats for trinucleotides, and ≥3 repeats for tetranucleotides, pentanucleotides, and hexanucleotides [40].The contraction and expansion of the IR regions were visualized using the IRscope online tool(https://irscope.shinyapps.io/irapp/) to investigate changes in the LSC/IRb/SSC/IRa boundary positions [41]. Phylogenetic analysis was performed using PhyloSuite_v1.2.3, with MAFFT alignment of *P. sinopubescens* and nine closely related species, followed by nucleotide diversity (Pi) analysis using DnaSP software (version 6.0). The window length and step size parameters were set to 600 and 200, respectively [42]. The Ka, Ks, and Ka/Ks ratios for the shared protein-coding genes (PCGs) across 10 *Polygonatum* species were extracted and calculated using CPStools and KaKs_calculator3 [43]. It is generally accepted that Ka/Ks < 1 indicates negative selection (purifying selection), meaning harmful mutations are eliminated, and the gene function is conserved. A Ka/Ks ratio of 1 suggests that the gene is in a neutral evolutionary state, with mutations not affected by natural selection. A Ka/Ks ratio > 1 suggests positive selection (adaptive evolution), where beneficial mutations are retained, aiding species adaptation to the environment.

### 2.5. Phylogenetic analysis

Phylogenetic analysis was conducted using the complete chloroplast genomes, with *Maianthemum* as the outgroup. Except for *P. sinopubescens*, all chloroplast genome sequences were retrieved from GenBank. The total sequence matrix was aligned using the MAFFT plugin in PhyloSuite v1.2.3, and the optimal substitution model was selected using ModelFinder based on the Bayesian Information Criterion (BIC). A maximum likelihood (ML) phylogenetic tree was reconstructed under the TVM + F + I + I + R3 model using IQ-TREE with 5000 ultrafast bootstrap replicates. The Bayesian (BI) phylogenetic tree was constructed using MrBayes under the GTR + I + G + F model. The phylogenetic tree was further refined using FigTree v1.4.4.

## 3. Results

### 3.1. Structure and characteristics of chloroplast group in *P. sinopubescens*

**Fig 2:**
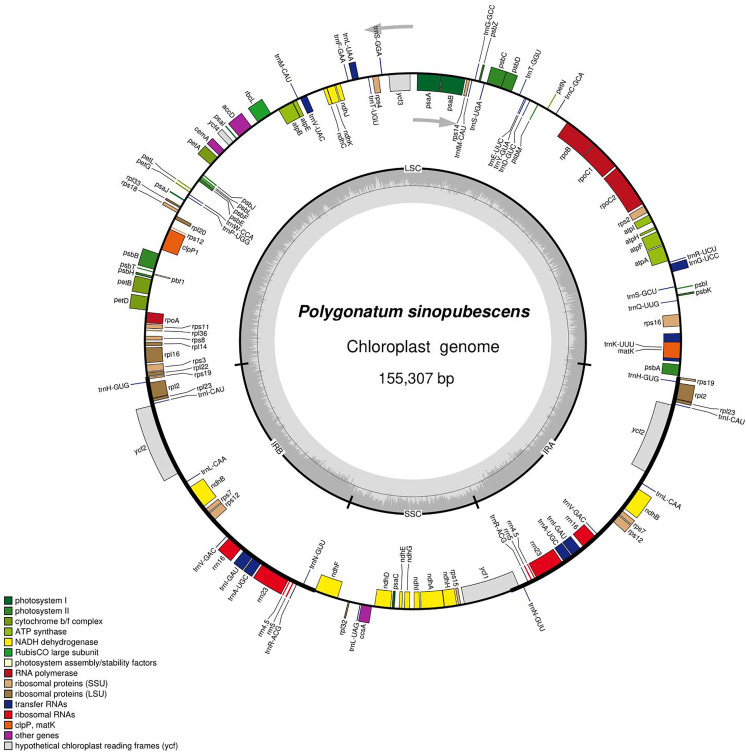
Gene map of *P. sinopubescens* chloroplast genome.

The *P. sinopubescens* chloroplast genome was a double-stranded circular molecule with a typical quadripartite structure, comprising a large single-copy region (LSC), a small single-copy region (SSC), and two inverted repeat regions (IRs). The total genome length was 155,307 bp (Fig 2), with an overall GC content of 37.68%. The LSC region was 84,252 bp in length with a GC content of 35.72%, the SSC region measured 18,455 bp with a GC content of 31.56%, and each IR region spanned 26,300 bp with a GC content of 42.98%. A total of 112 genes were annotated, including 78 protein-coding genes, 30 tRNA genes, and 4 rRNA genes, These genes are primarily involved in photosynthesis and self-replication. Among them, 11 genes contain introns, with 7 genes containing one intron and 4 genes containing two introns (Table 1).

**Table 1:** Chloroplast genome gene classification of *P. sinopubescens.*

Category	Gene groups	Gene names
Photosynthesis	Subunits_of_photosystem_I	psaA, psaB, psaC, psaI, psaJ
	Subunits_of_photosystem_II	pbf1, psbA, psbB, psbC, psbD, psbE, psbF, psbH, psbI, psbJ, psbK, psbL, psbM, psbT, psbZ
	Subunits_of_NADH_dehydrogenase	ndhA^*^, ndhB^**, (×2)^, ndhC, ndhD, ndhE, ndhF, ndhG, ndhH, ndhI, ndhJ, ndhK
	Subunits_of_cytochrome_b/f_complex	petA, petB^*^, petD^*^, petG, petL, petN
	Subunits_of_ATP_synthase	atpA, atpB, atpE, atpF^*^, atpH, atpI,
	Large_subunit_of_Rubisco	rbcL
Self-replication	Large_subunits_of_ribosome	rpl14, rpl16^*^, rpl2^**,(×2)^, rpl20, rpl22, rpl23^(×2)^, rpl32, rpl33, rpl36
	Small_subunits_of_ribosome	rps11, rps12^(×2)^, rps14, rps15, rps16^*^, rps18, rps19^(×2)^, rps2, rps3, rps4, rps7^(×2)^, rps8
	DNA-dependent_RNA_polymerase	rpoA, rpoB, rpoC1^*^, rpoC2
	Ribosomal_RNAs	rrn16^(×2)^, rrn23^(×2)^, rrn4.5^(×2)^, rrn5^(×2)^
	Transfer_RNAs	trnA-UGC^(×2)^, trnC-GCA, trnD-GUC, trnE-UUC, trnF-GAA, trnG-GCC, trnG-UCC, trnH-GUG^(×2)^, trnI-CAU^(×2)^, trnI-GAU^(×2)^, trnK-UUU, trnL-CAA^(×2)^, trnL-UAA^(×2)^,trnL-UAG, trnM-CAU, trnN-GUU^(×2)^, trnP-UGG, trnQ-UUG, trnR-ACG^(×2)^, trnR-UCU^(×2)^, trnS-GCU, trnS-GGA, trnS-UGA, trnT-GGU, trnT-UGU, trnV-GAC^(×2)^, trnV-UAC^(×2)^, trnW-CCA, trnY-GUA,trnfM-CAU
Other genes	Maturase	matK
	Protease	clpP1^**^
	Envelope_membrane_protein	cemA
	Acetyl-CoA_carboxylase	accD
	C-type_cytochrome_synthesis_gene	ccsA
	Translation_initiation_factor	
	protochlorophillide_reductase_subunit	
Genes of unknown	Proteins_of_unknown_function	ycf1, ycf2^(×2)^, ycf3^**^, ycf4

Note: *gene with a single intron; **gene with two introns; (×2) duplicated gene.

### 3.2. simple repeat sequence

**Fig 3:**
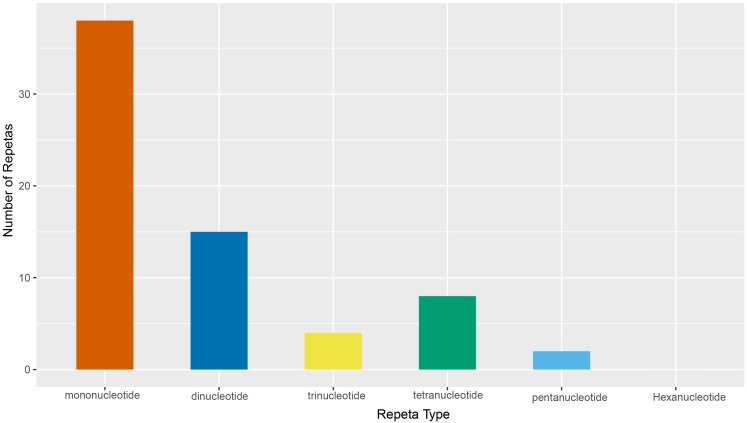
Simple sequence repeat types of *P. sinopubescens.*

Simple sequence repeats (SSRs) in the chloroplast genome of *P. sinopubescens* were detected using the MISA software (version 1.0) tool. The results showed (Fig 3) that a total of 67 SSR sequences were identified, categorized into 5 types. Among these, the most abundant were mononucleotide SSRs, with 38 sequences, accounting for 56.72% of the total; followed by 15 dinucleotide SSRs, which accounted for 22.39%; trinucleotide, tetranucleotide, and pentanucleotide SSRs numbered 4, 8, and 2, respectively, with proportions ranging from 0% to 11.94%. For mononucleotide repeats, A and T repeats dominated, comprising 97.37% of the total; dinucleotide repeats were predominantly AT/TA (80%). In trinucleotide SSRs, repeats composed of A and T bases (such as AAT and ATT) accounted for 75% of the total trinucleotide SSRs. Additionally, the LSC region contained the most SSR sequences, representing 76.12% of all SSRs. The REPuter-generated results indicated the identification of 59 dispersed repeat sequences, including 7 complementary repeat sequences (C) and 52 palindromic repeat sequences (P).

### 3.3. Codon usage frequency analysis

**Fig 4:**
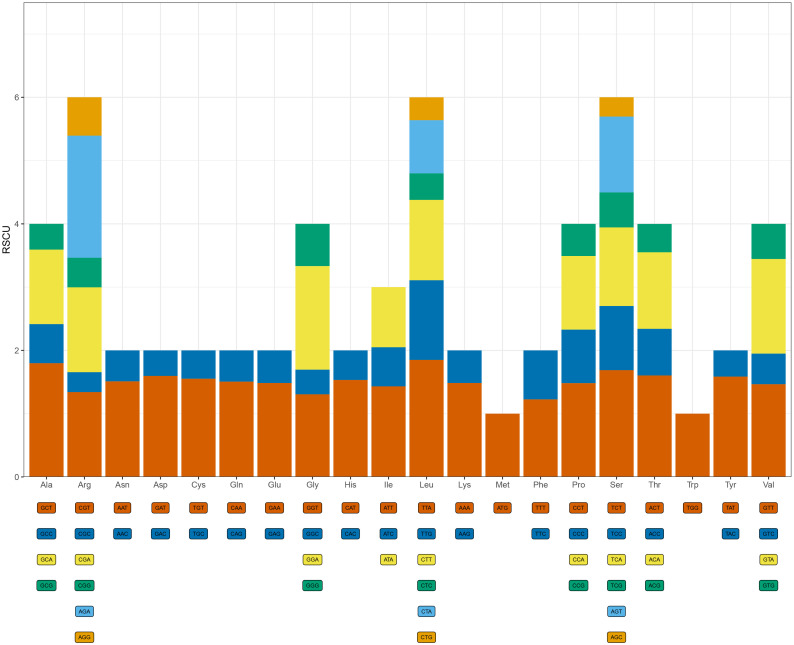
Codon usage graph of *P. sinopubescens.* Note: The horizontal coordinate is the amino acid encoded by the codon, and the vertical coordinate is the RSCU value.

Based on the protein-coding genes of the complete chloroplast genome, the codon usage frequency in *P. sinopubescens* was calculated. A total of 61 codons encoding 20 amino acids. Among these, leucine (Leu) was the most frequently used amino acid, with a total of 2,673 occurrences, followed by isoleucine (Ile) and serine (Ser), with 2,267 and 2,048 occurrences, respectively. Cysteine (Cys) was the least frequently used amino acid, with only 304 occurrences. The most frequently used synonymous codon was ATT, encoding isoleucine (Ile), with 1,082 instances (4.14%), while the least frequently used codon was TGC, encoding cysteine (Cys), with only 68 instances (0.26%). Codon usage bias was analyzed using Cusp (EMBOSS v6.6.0.0) software to calculate the Relative Synonymous Codon Usage (RSCU) values. High codon usage bias was detected for 30 codons with an RSCU > 1, while low codon usage bias was observed for 29 codons with an RSCU < 1. These results indicate that the chloroplast genome of *P. sinopubescens* exhibits a significant codon usage bias. Additionally, no codon usage bias was detected for methionine and tryptophan (RSCU = 1), and the third position of all highly preferred codons (RSCU > 1) primarily included 28 A/T codons (Fig 4).

### 3.4. IR contraction and expansion

**Fig 5:**
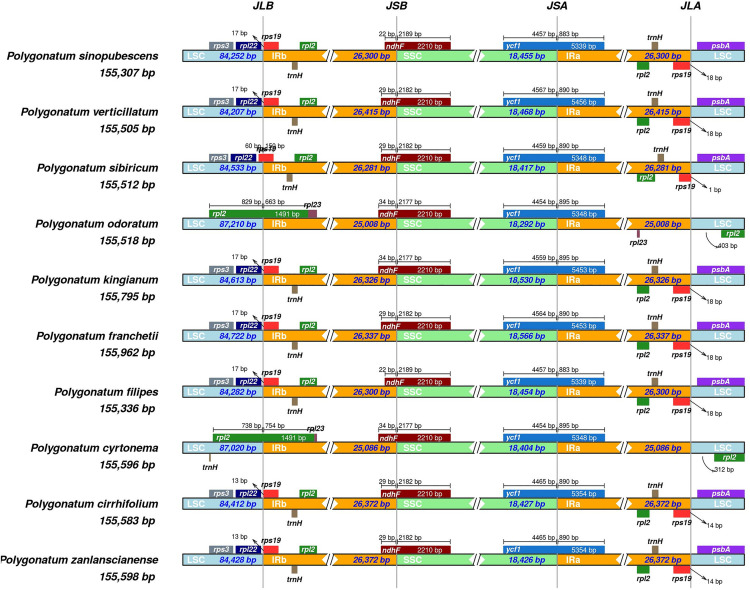
Comparison of boundaries regions of *Polygonatum* chloroplast genome.

The contraction and expansion of the IR regions reveal structural variations at the LSC/IR/SSC junctions. Using the IRscope online program, we analyzed the expansion and contraction of the IR regions in the chloroplast genomes of 10 *Polygonatum* species (Fig 5). The results showed that the length of the IR regions was relatively conserved, ranging from 25,008 bp in *P. odoratum* to 26,415 bp in *P. sibiricum*, and the gene content at the IR/SC boundaries was generally consistent. The genes *rpl*22, *rps*19, *ndh*F, *ycf*1, and *psb*A were located at the IR boundary regions, and significant differences were observed in the contraction and expansion of the IR regions. In most *Polygonatum* species, the *rps*19 gene was entirely located in the IRb region, positioned 13 bp or 17 bp from the IRb boundary. In contrast, *rps*19 was absent in *P. cyrtonema* and *P. odoratum*, which may be attributed to the extended length (1,492 bp) of the *rpl*2 gene that bridges the LSC and IRb regions, with distances of 754 bp and 663 bp from the IRb boundary, respectively. The *ndh*F gene spanned the junction between IRb and SSC, with 22–34 bp located within the IRb region. The *ycf*1 gene formed the junction between SSC and IRa, and its distance from the IRa boundary ranged from 883 bp to 895 bp. In addition, *rpl*2 and *trn*H were present in the IRa region, while *psb*A was located in the LSC region.

### 3.5. Nucleotide diversity analysis and selective pressure

**Fig 6:**
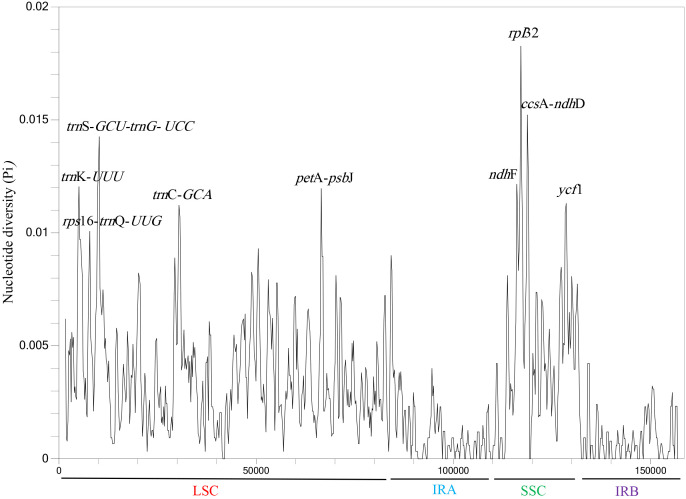
Nucleotide diversity (Pi) analysis of chloroplast genomes in *Polygonatum* species with 600 bp sliding window length and 200 bp step size.

Nucleotide diversity (Pi) of ten chloroplast genes was analyzed using DnaSP software (version 6.0) to identify mutational hotspot regions in the chloroplast genomes of *Polygonatum* species. The results showed that nine regions had Pi values greater than 0.010, namely *trn*K-*UUU*, *rps*16-*trn*Q-*UUG*, *trn*S-*GCU*-*trn*G-*UCC*, *trn*C-*GCA*, *pet*A-*psb*J, *ndh*F, *rpl*32, *ccs*A-*ndh*D, and *ycf*1 (Fig 6). These high-Pi regions represent potential divergence loci within the chloroplast genomes of the ten Polygonatumspecies analyzed. Among them, five mutational hotspots (*trn*K*-UUU*, *rps*16*-trn*Q-*UUG*, *trn*S-*GCU*-*trn*G-*UCC*, *trn*C-*GCA*, *pet*A-*psb*J) were located in the LSC region, while four (*ndh*F, *rpl*32, *ccs*A-*ndh*D, *ycf*1) were located in the SSC region. The *rpl*32gene fragment, located in the SSC region, exhibited the highest level of variation, with a coefficient of 0.01826. Notably, no highly variable sites were detected in the IR regions, further supporting the high conservation of the IR regions in the chloroplast genomes of *Polygonatum* species. These nine high-Pi sequences can serve as potential DNA markers for elucidating genetic differentiation among different taxa within the genus *Polygonatum*.

**Fig 7:**
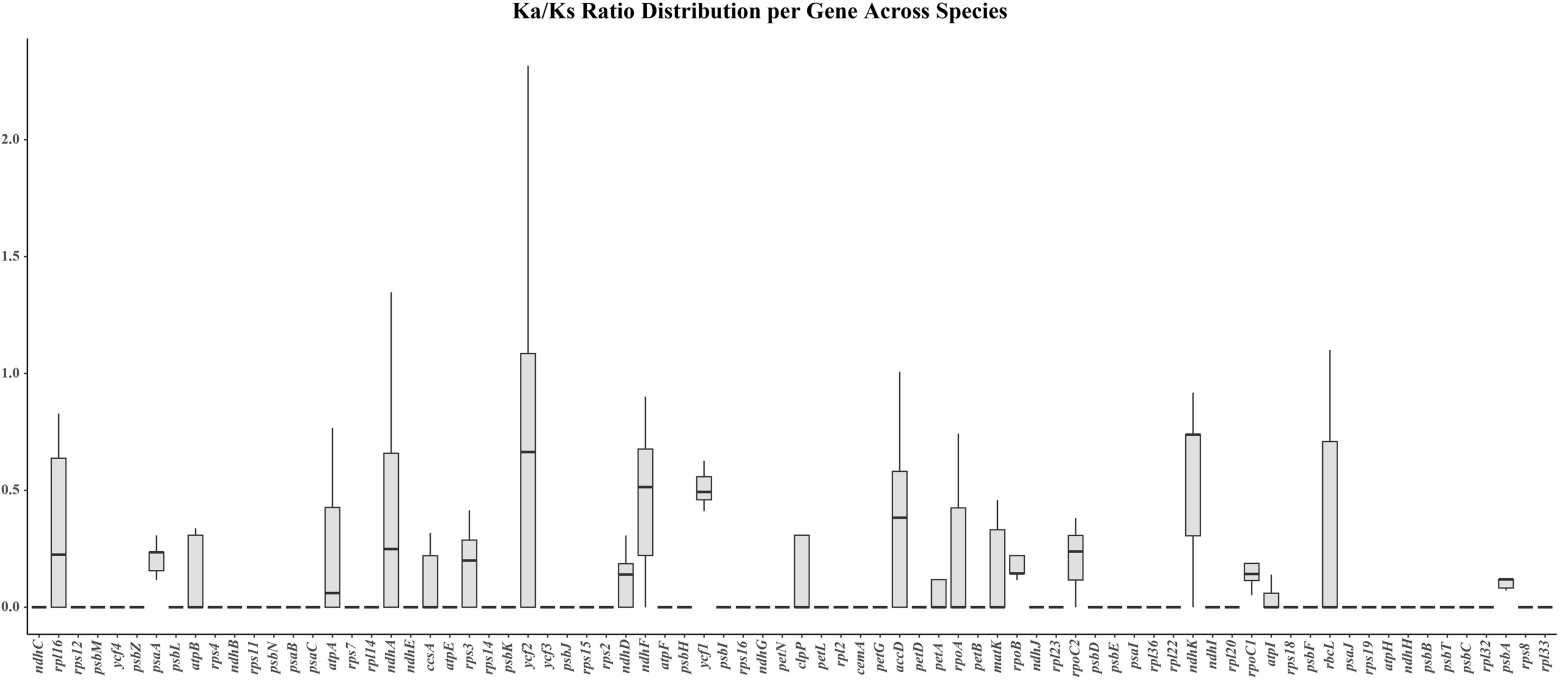
The Ka/Ks ratio of 78 common protein-coding genes.

To investigate the molecular evolutionary processes of chloroplast protein-coding genes in *Polygonatum*, we estimated the ratio of nonsynonymous (Ka) to synonymous (Ks) substitutions using 78 shared protein-coding genes (CDS) for selection pressure analysis (Fig 7). The results showed that the Ka/Ks values of most genes were lower than 1, indicating that these protein-coding genes have been subjected to strong purifying selection. Only a very small number of genes exhibited Ka/Ks > 1 (including *ndh*A, *ycf*2, *acc*D, and *rbc*L), and this pattern was observed only in a few species. Among them, the *ycf*2 gene showed the highest Ka/Ks value in *P. verticillatum* (2.31676), followed by *P. sibiricum*(2.00985) and *P. kingianum* (1.08535). The *ndh*A gene exhibited a Ka/Ks value of 1.34745 in *P. verticillatum*, the *acc*D gene had a Ka/Ks value of 1.00734 in *P. kingianum*, and the *rbc*L gene showed Ka/Ks values of 1.8394 in *P. franchetii* and 1.1006 in *P. sibiricum*. These genes exhibited relatively high substitution rates and evolutionary rates in specific species, suggesting evidence of positive selection. Functionally, the positively selected genes can be classified into photosynthesis-related genes (e.g., *ndh*A, *rbc*L) and other functional categories (e.g., *ycf*2, *acc*D), indicating that most of the genes under positive selection are closely associated with the photosynthetic system.

### 3.6. Phylogenetic analysis

**Fig 8:**
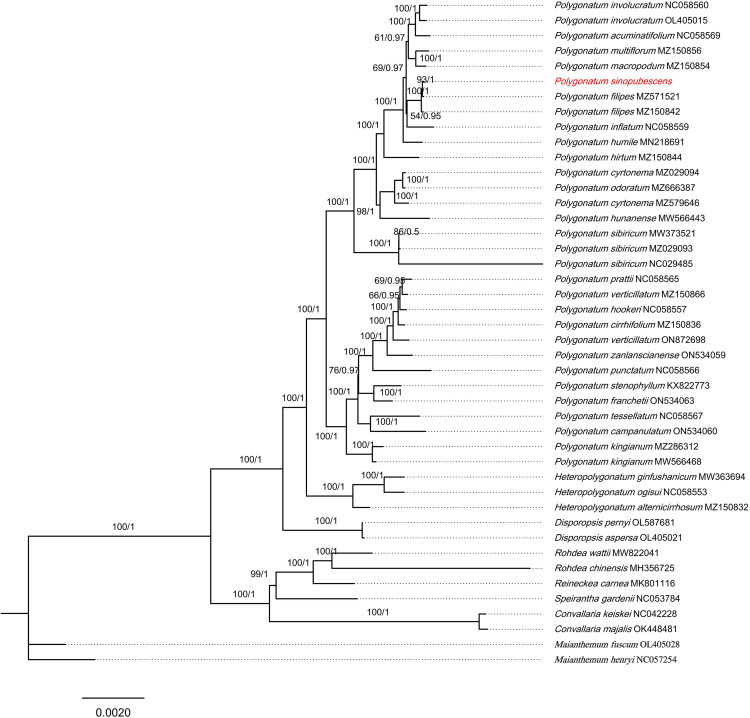
Maximum likelihood (ML) and Bayesian inference (BI) methods were used to reconstruct the tree. Only ML tree was shown, because of the highly identified topologies of ML tree and BI tree. The value of ML supports and Bayesian posterior probabilities were shown above the branches. The cp genomes newly sequenced in this study are highlighted with red font marks.

Chloroplast genomes are widely employed in phylogenetic analyses across diverse plant taxa. To clarify the phylogenetic position of *Polygonatum* species, *Maianthemum henryi* (Baker) LaFrankie and *Maianthemum fuscum* (Wall.) LaFrankie were selected as outgroups. A total of 44 complete chloroplast genome sequences were used to construct phylogenetic trees using Maximum Likelihood (ML) and Bayesian Inference (BI) methods. The ML and BI trees exhibited congruent topologies (Fig 8), with most nodes receiving strong statistical support, thereby confirming the monophyly of *Polygonatum*, *Maianthemum*, and *Disporopsis*. *Polygonatum* was resolved as a sister clade to *Heteropolygonatum* (BS = 100; PP = 1). Within *Polygonatum*, three major clades were identified: sect. *Verticillata*, sect. *Polygonatum*, and sect. *Sibirica* (BS = 100; PP = 1), with sect. *Sibirica* comprising only a single species, *P. sibiricum*. *P. sinopubescens* and *P. filipes* formed a distinct and strongly supported clade (BS = 100; PP = 1), indicating a close evolutionary relationship. Furthermore, the pharmacopoeial species *P. odoratum*, P. *cyrtonema*, *P. sibiricum*, and *P. kingianum*, which are listed in the Chinese Pharmacopoeia, were clearly distinguishable from other *Polygonatum* species with high support values, underscoring their distinct genetic identities.

## 4. Discussion

### 4.1. Characteristics analysis of the chloroplast whole genome

In this study, the complete chloroplast genome of *P. sinopubescens* was analyzed. The genome exhibits a typical quadripartite structure, consisting of a large single-copy (LSC) region, a small single-copy (SSC) region, and two inverted repeat (IR) regions forming a circular double-stranded molecule. The total genome length is 155,307 bp, which is comparable to previously reported chloroplast genomes of other *Polygonatum* species [44]. The chloroplast genome displays a higher AT content than GC content, with the GC content in the IR regions being higher than that in the LSC and SSC regions. Our results indicate that the total length, GC content, and gene composition of the *P. sinopubescens* chloroplast genome are nearly identical to those of other *Polygonatum* species [42]. In total, the chloroplast genome of *P. sinopubescens* encodes 112 genes, including 78 protein-coding genes, 30 tRNA genes, and 4 rRNA genes. Among them, 11 genes contain introns, and 22 genes are located within the IR regions.

Simple sequence repeats (SSRs) are an important class of codominant DNA molecular markers that have been widely used in species identification, phylogeography, and population genetics due to their high abundance, random distribution in genomes, and rich polymorphism information [45–47]. In this study, a total of 67 SSRs were detected, among which mononucleotide SSRs were the most frequent in all genomes and were predominantly composed of A/T motifs, accounting for 97.37% of the total. Dinucleotide repeats ranked second in abundance, with AT/TA motifs being the most common, representing 80% of this category. These results indicate that SSRs in the chloroplast genomes of *Polygonatum* species are strongly biased toward A and T bases, consistent with findings from other *Polygonatum* taxa [42,44,48] and similar to the SSR composition observed in the chloroplast genomes of most angiosperms [49–52]. SSRs rich in A/T have higher mutation rates and are more likely to generate polymorphic loci, making them suitable as high-resolution genetic markers. The cpSSRs identified in this study hold promise as valuable molecular marker resources for *Polygonatum* species identification, genetic diversity assessment, and phylogenetic studies.

As the link between nucleic acids, proteins, and genetic material, codons play a crucial role in the transmission of genetic information and provide reliable insights into gene function and species evolution [53,54]. In this study, a total of 61 codons were identified, among which leucine (Leu) was the most frequently encoded amino acid, followed by isoleucine (Ile) and serine (Ser). The most frequently used codons for these amino acids are TTA, ATT, and TCT. Previous studies have demonstrated that GC content is closely associated with mutational pressure or natural selection, whereas interspecific differences in codon usage frequency may be related to evolutionary status, ecological environment, and nucleotide composition [55]. Elucidating the characteristics of codon bias and its variation is of great significance for advancing our understanding of molecular evolution and the biodiversity of heterologous gene expression across species [56,57]. Based on the relative synonymous codon usage (RSCU) analysis, most high-frequency and highly expressed codons ended with A or U, further supporting the A/U bias at the third codon position in medicinal *Polygonatum* species. Moreover, SSR analysis revealed a pronounced preference for A and T nucleotides. Given that A/T base pairs, which form two hydrogen bonds, are more easily disrupted than G/C base pairs, the preference for A and T nucleotides in the chloroplast genomes of *Polygonatum* species may contribute to their strong adaptive capacity and pronounced structural variation in response to environmental changes. However, the underlying mechanisms behind this phenomenon require further investigation.

### 4.2. Comparative analysis of chloroplast genomes

The contraction or expansion of IR/SC boundaries is a major driver of chloroplast genome size variation. In *Polygonatum*, the boundary genes are primarily *rpl22*, *rps19*, *ndhF*, *ycf1*, and *psbA*, which is consistent with previous studies on this genus [58], suggesting that boundary characteristics are relatively conserved among closely related species [59]. Studies of chloroplast genomes in monocotyledonous plants have shown that the *rps19* gene is located in the IR region [60]. In *Polygonatum*, many *rps19* genes are entirely located within the IR region [46,61]; however, in this study, we found that the *rps19* gene of *P. sibiricum* was partially located in the LSC region, which may be attributed to IR contraction. In *P. cyrtonema* and *P. odoratum*, the *rps19* gene was missing, which may have resulted from the elongation of the *rpl2* gene, thereby bridging the LSC and IRb regions. The stability of the IR/SC boundary suggests that *Polygonatum* species may have experienced relatively low selective pressure during evolution, consistent with their broad ecological adaptability and strong species differentiation ability [62].

Evaluation of Ka/Ks values for protein-coding genes containing RNA editing sites can provide insights into functional diversity, structural variation, and evolutionary processes. The Ka/Ks ratio is commonly used to determine whether protein-coding genes are subject to selective pressure and has been widely recognized as a key metric for assessing adaptive evolutionary rates and positive selection. Our selective pressure analysis indicated that most genes have undergone purifying selection, consistent with a pattern of conservative evolution. Notably, *ndhA*, *ycf2*, *accD*, and *rbcL* exhibited signatures of positive selection. Among these, *ndhA* and *rbcL* are photosynthesis-related and systemic genes, respectively. Given that *Polygonatum* species predominantly grow on shaded forest slopes, in thickets, or under canopies, their adaptation to light stress may represent an important genetic basis for chloroplast genome evolution in this genus [58].

Nucleotide diversity analysis revealed that the highly variable regions of the *Polygonatum* chloroplast genome were mainly located in the LSC and SSC regions. Nine hypervariable Pi fragments were identified: *trnK-UUU*, *rps16-trnQ-UUG*, *trnS-GCU-trnG-UCC*, *trnC-GCA*, *petA-psb*J, *ndhF*, *rpl32*, *ccsA-ndhD*, and *ycf1*. These mutational hotspots provide potential chloroplast DNA barcode references for the molecular identification of *Polygonatum* species in future studies.

### 4.3. Phylogenetic analysis

The phylogeny and classification of the genus *Polygonatum* have long been controversial. In this study, a phylogenetic tree was reconstructed based on complete chloroplast genome sequences. The results provided strong support for the monophyly of *Polygonatum* (Fig. 8), with *Polygonatum* and *Heteropolygonatum* resolved as sister clades. Within *Polygonatum*, three well-supported clades were identified: sect. *Polygonatum*, sect. *Sibirica*, and sect. *Verticillata*, with sect. *Verticillata* representing a relatively ancestral lineage within the genus. This finding is consistent with the results of Shi Naixing [53].The phylogenetic tree confirmed the systematic position of *P. sinopubescens* within *Polygonatum*, showing that *P. sinopubescens* and *P. filipes* form a sister group within sect. *Polygonatum*. This relationship is supported by previous morphological, cytological, and molecular evidence [35,63,64], further corroborating the close phylogenetic relationship between the two species. Additionally, strong support was found for the monophyly of *P. sibiricum*, consistent with findings from other studies [65–67].In summary, our study enriches the genomic resources of *Polygonatum* and provides valuable insights into the phylogenetic relationships within the genus. The findings on *P. sinopubescens* also have important implications for the exploration and conservation of *Polygonatum* genetic resources.

## Supporting information

S1 File The new dataset generated by this study has been included in the supplementary materials, while the other datasets are all from the following public domain resources: https://www.ncbi.nlm.nih.gov/#!/edu/home/principal/inicio. (ZIP)
